# C-Reactive Protein and Cancer: Interpreting the Differential Bioactivities of Its Pentameric and Monomeric, Modified Isoforms

**DOI:** 10.3389/fimmu.2021.744129

**Published:** 2021-09-06

**Authors:** Lawrence A. Potempa, Ibraheem M. Rajab, Margaret E. Olson, Peter C. Hart

**Affiliations:** College of Science, Health and Pharmacy, Roosevelt University Schaumburg, Schaumburg, IL, United States

**Keywords:** CRP isoforms, mCRP, cancer, inflammation, C-reactive protein, anti-cancer, innate immunity

## Abstract

C-reactive protein (CRP) was first recognized in the 1940s as a protein that appeared in blood during acute episodes of infectious disease. Its presence and pharmacodynamics were found in essentially all diseases that involved tissue damage and inflammation. Identified as a major component of the innate, unlearned immunity, it became a useful diagnostic marker for the extent of inflammation during disease exacerbation or remission. Efforts to define its true biological role has eluded clear definition for over a half-century. Herein, a unifying concept is presented that explains both pro-inflammatory and anti-inflammatory activities of CRP. This concept involves the recognition and understanding that CRP can be induced to undergo a pronounced, non-proteolytic reorganization of its higher-level protein structures into conformationally distinct isomers with distinctive functional activities. This process occurs when the non-covalently associated globular subunits of the pentameric isoform (“pCRP”) are induced to dissociate into a monomeric isoform (“mCRP”). mCRP consistently and potently provides pro-inflammatory activation and amplification activities. pCRP provides weak anti-inflammatory activities consistent with low-level chronic inflammation. mCRP can spontaneously form in purified pCRP reagents in ways that are not immediately recognized during purification and certification analyses. By now understanding the factors that influence pCRP dissociate into mCRP, many published reports investigating CRP as a biological response modifier of host defense can be reevaluated to include a discussion of how each CRP isoform may have affected the generated results. Specific attention is given to *in vitro* and *in vivo* studies of CRP as an anti-cancer agent.

## Introduction

C-reactive protein (CRP) is an evolutionarily conserved multimeric protein of innate immunity. In humans, its blood levels change acutely and quantitatively in response to the severity of tissue damage and the inflammatory response that ensues. The immediate, natural response to tissue damage is referred to as the acute phase response (APR) which is a natural biochemical, physiological, and immunological reaction to the tissue insult. The APR is an amnestic response with the multifaceted purpose of controlling vascular damage, accessing, accumulating, and amplifying host humoral and cellular defenses, controlling any pathology associated with the introduction of the thereat, removing debris and stimulating tissue repair mechanisms to reestablish healthy tissue homeostasis. These processes can also stimulate adaptive immune responses which can confer longer term immunological memory (1, 2).

CRP is widely called the prototypic acute phase reactant. As a protein of innate immunity, one defined function is as a pattern recognition receptor with each of its five homologous globular subunits having a calcium-regulated binding pocket for ligands expressing phosphocholine (PC) moieties. In addition to binding PC-containing teichoic acid in Gram positive bacteria, CRP can also bind to activated cell membranes in which PC groups on phospholipids become accessible when diacyl phospholipids get hydrolyzed into monoacylphospholipids [Lyso-phosphatidyl choline (Lyso-PtC)]. When liganded, bound CRP can activate endothelial cells, platelets and leukocytes and the complement system and influence the overall inflammatory response that results from the inciting threat to homeostasis. As a key protein of the APR, its role in activating and/or regulating any of the host defense processes that are part of innate immunity has been widely studied (3–6).

Over many decades, there have been conflicting conclusions as to the true nature of CRP’s bioactivities. One explanation for confounding conclusions is that CRP has long been assumed to be a single structural protein (i.e., a cyclic pentameric discoid protein) that remains unchanged by the biochemical forces at work in the various acute phase and inflammatory processes being studied. Over the more recent decades, however, numerous studies have shown that CRP is, in fact, a protein that can undergo a non-proteolytic subunit dissociation which results in a conformational change into at least two structurally and functionally distinctive isoforms. When dissociated, the serum soluble diagnostically useful pentameric, non-glycosylated protein (referred to as “pentameric” or “pCRP”) is converted into an antigenically distinct isoform that has reduced aqueous solubility such that it can partition into cholesterol-rich membrane lipid rafts (referred to as “modified-monomeric” or “mCRP”) (5, 7). Of underappreciated significance, mCRP can form from purified pCRP spontaneously with storage or by exposing pCRP to surfaces that contribute apolar binding sites (8). By carefully defining and differentiating the structural and bioactivities of pCRP and mCRP (summarized in **Table 1**), and by understanding the extent by which mCRP can be expressed in a stored pCRP solution, the distinctive bioactivity of each of pCRP and mCRP isoforms can be compared. It is now established that pCRP has a weak anti-inflammatory bioactivity while mCRP has a strong pro-inflammatory bioactivity. By focusing on this important distinction, and by carefully evaluating the methods used in describing published studies on *in vitro* CRP bioactivities, a consistent explanation emerges on the role which CRP has in providing natural defense against any threats to tissue integrity. This report reviews and elaborates on previous studies looking into putative roles for “CRP” in cancer-related host defense immune responses by describing how conclusions drawn prior to the appreciation of the mCRP isoform may now be interpreted in terms of how mCRP may have contributed to reported biological effects.

**Table 1:** Comparison of Biochemical Characteristics of Pentameric (pCRP) and modified, monomeric (mCRP) C-reactive protein.

Characteristic	Pentameric CRP (pCRP)	Modified, monomeric CRP (mCRP)
**Size**	➢ Mr 116, 145 ➢ 5 non-glycosylated identical globular subunits, non-covalently associated in cyclic symmetry ➢ Flat discoid shape with a central void ➢ Each subunit contains 2 bound calcium ions and one binding pocket for Phosphocholine (PC) ➢ All PC binding pockets orient to one face of the disc; the opposite face contains a helical segment that interacts with other ligands involving effector responses ➢ Average diameter by EM 10.42 ± 0.08	➢ Mr 23,229 ➢ Free subunit that is conformationally distinct (relaxed) from that subunit found in pCRP ➢ Isolated protein has reduced aqueous solubility and will self-aggregate into multimers as a function of ionic strength and presence of divalent cations ➢ Average diameter by EM |14.425 ± 0.33
**Electrophoretic Mobility**	Gamma	Alpha
**Isoelectric Point**	6.4	5.4
**Stroke’s Radius**	40 ± 5 Å	30 ± 4 Å
**Solubility**		
**@ 0.15 ionic strength**	> 1 mg/ml	< 100 ug/ml
**@ 0.015 ionic strength**	Decreased	> 600 ug/ml
**Calcium effect**	➢ Stabilizes (compacts) pentamer quaternary structure ➢ Confers resistance to proteolysis ➢ Regulates structures that allows for binding to PC ligand	➢ Calcium removal facilitates spontaneous dissociation of the pentamer and expression of the mCRP isoform ➢ Once formed, calcium and other divalent cations will lead to protein aggregation and precipitation
**Reported Bioactivities**	➢ Opsonizes pathogens ➢ Binds perturbed membranes where PC groups become exposed ➢ Neutralizes PAF-induced neutrophil degranulation and RO production ➢ Scavenges for/facilities removal of nucleic acid/chromatin cell debris ➢ Activates classical complement pathway ➢ Regulates alternative complement pathway activation by binding Factor H ➢ Stimulate leukocyte phagocytosis and oxidative metabolism ➢ Is weakly ant-inflammatory	➢ Increases P-selection expression ➢ Increases platelet activation and aggregation with neutrophils ➢ Increases leukocyte adhesion to endothelial cells, synthesis and release of IL-8 synthesis and MCP-1 ➢ Augments respiratory burst response ➢ Delays apoptosis ➢ Stimulates cytokine release ➢ Increases ICAM-1, VCAM-1, and E-Selection expression on endothelialcells ➢ Activates classic complement and inhibits alternative complement pathway activation

References: 4, 5 and 8–21.

## Diagnostic Relevance of CRP Measurement in Cancer

As with any medical condition involving a host defense response to tissue damaging pathologies, blood levels of CRP are used to monitor the presence and extent of inflammation in cancer patients (6). Most generally, blood values reflect on the pCRP isoform, which is serum soluble and easily measured using nephelometric and turbidimetric assays. Because CRP levels can increase 100-500-fold in 24-72 hrs. and can decrease to baseline quickly with a 19-hr. half-life (22), the relative usefulness of CRP as a diagnostic tool is often difficult to assess as its relevance is intimately tied to the exact time and immediate clinical presentation of the patient at the time a blood sample was drawn. This fact, along with the evolving understanding that pCRP can be converted into mCRP *in situ*, which is not, at present, easily quantified in aqueous blood samples, have limited interpretation of the meaning of CRP as an index of inflammation in cancer patients. Further complicating the diagnostic interpretation of CRP is the introduction of high-sensitivity CRP values (i.e., hsCRP) which are described as CRP values less than or equal to 10 µg/ml. While baseline levels of CRP are described at 1-3 µg/ml, and diagnostically relevant blood levels as described by FDA guidance are ≥ 10 µg/ml, hsCRP levels have been purported in many reports to describe either or both a micro-inflammation condition and a predictive criterion for future disease (6, 23).

Our group has published two recent reviews (6, 7) that specifically address both the diagnostic interpretation of and the biological activities of pCRP, hsCRP and mCRP in cancer. Readers are directed to these reviews for details, developments, and cross references. In brief, these reviews present evidence that

1. serum pCRP levels more readily reflect on the state of tissue damage and disease involvement (e.g., reflective of cancerous growth) in a patient rather than ongoing inflammation;
2. hepatically produced pCRP will, in the first hours of an inciting cause, localize to the affected tissue site where it will dissociate and conformationally change into the mCRP isoform which will enter into membrane lipid rafts and stimulate an aggressive pro-inflammatory response;
3. during these first hours, blood levels of pCRP remain low or undetectable;
4. as pCRP continues to be produced and released by the liver, the rate of conversion from pCRP to mCRP slows down, shifting an acute inflammatory response to a chronic inflammatory response at the site of tissue involvement;
5. circulating blood pCRP levels increase in proportion to the continued level of tissue damage ongoing at the site(s) of disease;
6. baseline pCRP levels are defined at 1-3 µg/ml;
7. blood pCRP levels above 10 µg/ml are indicative of ongoing, concerning tissue damage associated with cancer disease;
8. pCRP levels above 50-100 µg/ml are prognostic of poor outcomes as this indicates extensive tissue involvement and disease progression;
9. as pCRP blood levels rapidly rise and fall, the time and the clinical state of disease when samples are drawn are critical parameters in assessing the diagnostic utility of pCRP measurements.

Monitoring pCRP levels during any treatment regimen is a reasonable and simple way to assess success and remission; measuring pCRP levels during remission can be diagnostic of long-term disease stability or an early sign of recurring cancerous growth. Because this protein can assume two distinctive conformations with contrasting bioactivities, CRP can play a role in both amplifying and dampening inflammation and host defense responses involved in the acute phase response. In this review, we describe previous studies of how “CRP” affected various acute phase and inflammation processes of relevance in cancer disease. By reinterpreting results to include concepts of mCRP, a more consistent understanding of how “CRP” contributes to the acute phase/inflammatory response in cancer, and in any disease involving tissue damage, is advanced (6, 7, 23).

## C-Reactive Protein Exists in Distinctive Conformational Forms With Different Solubility, Biodistribution and Bioactivity

Numerous reports have now appeared describing the structural and functional distinctiveness of conformational isoforms of CRP (5–7, 21, 24, 25). Current evidence describes at least three isoforms of CRP, 1.) the soluble non-covalently associated pentameric discoid protein, synthesized and secreted by hepatocytes in response to IL-6 stimulation and quantifiable in serum (pCRP), 2.) the poorly soluble, lipid raft inserting modified, monomeric isoform (mCRP), and 3.) a transitional intermediate that forms when pCRP binds to membrane exposed PC groups and expresses some mCRP structural and antigenic characteristics while still in a pentameric conformation (referred to as mCRP_m_ or pCRP*). In appreciation of the biochemical processes and energies involved in the dissociation of pCRP into mCRP, it is apparent that mCRP can spontaneously form by surface denaturation in purified pCRP reagents (8). Because the mCRP formation does not involve proteolysis and would migrate in SDS PAGE with the same molecular weight as pCRP subunits (i.e., ~ 23kDa), and, while mCRP has distinct antigenicity from pCRP, all polyclonal anti-CRP reagents evaluated to date appear to contain specificity for both pCRP and mCRP antigens. Although mCRP has much reduced aqueous solubility (see **Table 1**), it, or its self-aggregate complexes, can exist to some quantitative level, in an isolated pCRP sample. In direct comparison studies with carefully prepared reagents, mCRP bioactivities appear to be approximately 10-100-fold more potent than pCRP (26, 27). Of relevance to published studies therefore, when “CRP” was added to experimental systems prior to the appreciation of the mCRP isoform, there is a likelihood that added protein may have included both mCRP and pCRP isoforms. As mCRP is more potent, even a small level of “contaminating” mCRP could produce measurable experimental responses. The relative level of mCRP in any isolated reagent would be expected to be influenced by length of storage, temperature of storage, admixing CRP with lipids, vortexing, the inclusion of calcium (which stabilizes the pCRP isoform), or chelator (EDTA, citrate) which accelerates the spontaneous conversion of pCRP into mCRP (See **Supplementary Figure 1**). With the understanding that distinctive isoforms of CRP exist and that such isoforms co-exist in purified CRP reagents, ambiguities to the real bioactivities of CRP in historically published studies can be explained. Below, focus is given to assays relevant to host defense responses to cancer disease.

## Differentially Interpreting the Effects of pCRP and mCRP in Models of Cancer

As recently summarized by Hart et al. (6), cancer patients are known to have elevated levels of blood CRP. Furthermore, patients with malignant disease have much higher levels of CRP than patients with benign disease, and the higher the CRP level (e.g., > 50-100 µg/ml), the worse the prognosis. Several *in vitro* and *in vivo* studies investigating a possible role for CRP as an agent that can affect cancer disease have appeared in the literature. Below, and in **Table 2**, these studies are summarized. In addition, and relevant to evolving understanding of distinctive CRP isoforms presented in this review, published results are reevaluated to explain measured CRP effects more consistently in terms of the pro-inflammatory mCRP isoform and the anti-inflammatory pCRP isoform.

**Table 2:** Activities of pCRP and mCRP with various Cancer models.

Cancer Type	CRP Reagent Used	Mechanistic Notes	References
**Melanoma**	Isolated CRP	72 hr. growth in tissue culture Added with lymphocytes Stopped growth of tumor cells	➢ (28)
**Fibrosarcoma**	CRP encapsulated in liposomes	Anti-metastatic, especially when injected early in disease course Non-encapsulated CRP was not similary effective	➢ (29)
**Melanoma Fibrosarcoma Sarcoma**	CRP encapsulated in liposomes	Showed tumoricidal activity Anti-tumor response found in exudate macrophages and involved enhanced oxidate metabolism	➢ (30)
**Colon Adenocarcinoma**	CRP encapsulated in liposomes	Treatment resulted in fewer and smaller liver metastases	➢ (31)
**Fibrosarcoma Colon carcinoma**	CRP encapsulated in liposomes	Stimulated peritoneal, exudate cells Required an intact complement system	➢ (32) ➢ (33)
**Mastocytoma** **Fibroblast carcinoma CAK-1 (mesothelial & ovarian) carcinomas**	Isolated CRP Heat aggregated CRP lost tumoricidal activity	CRP added to tissue cultured cells CRP affect did not involve lymphokines CRP-activated macrophages did not affect normal fibroblast	➢ (34)
**Astrocytoma Renal carcinoma Melanoma**	Isolate CRP	CRP activated peripheral blood mononuclear cells (PBMs) but required incubation on glass–adherent cells for 20-44 hr. Tumoricidal activity involved superoxide anion generation Normal PBMs were similarly activated by CRP but normal fibroblasts and glial cells were not	➢ (35, 36)
**Fibrosarcoma**	Peptides derived from CRP in liposomes	Identified a peptide with anti-tumor and anti-lung metastatic activity Peptide 174IYLGGPFSPNVL185 Stimulated Peripheral blood MAC 1+ (CR3+) cells Required encapsulated in a liposome	➢ (37, 38)
**Fibrosarcoma**	CRP encapsulated in liposome CRP-peptide encapsulated in liposomes	Increased IL-1 and TNF secretion from monocytes and alveolar macrophages When IL-2 was co-administered with CRP regeant, reported enhanced Influenced IL-1-β, IL-6 and TNF-α secretion on cultured monocytes	➢ (39–41) ➢ (42) ➢ (43)
**Breast Adenocarcinoma**	mCRP in LUVETS CRP in LUVETs Isolated pCRP Isolated mCRP	mCRP alone or in LUVETS slowed tumor growth, caused necrosis at site of tumor growth and reduced metastasis pCRP alone or in LUVETS was ineffective in slowing tumor growth or preventing metastasis	➢ (44, 45)
**Lewis Lung Carcinoma CAPAN-2 Pancreatic**	mCRP Recombinant nCRP	Slower tumor growth Cased necrosis at tumor site Anti-cancer effects observed in nude mice	➢ See **Supplementary Figure 2** ➢ (8)

### Human Melanoma

Hornung (28) first studied the effect of CRP on cultured human melanoma cells. While CRP did not directly alter the growth curve of such cells, CRP added with lymphocytes not only resulted in no growth of the melanoma cells after 72 hours of culture but resulted in a 75% reduction in viability of the initial inoculum. The CRP used in these studies was reported to be toxic to the lymphocytes themselves while still somehow being able to contribute to anti-melanoma activity. It is now established that adding pCRP to tissue culture cells causes pCRP to convert into mCRP within the first four hours after addition of pCRP (11, 46–48). Hence, the three-day culture used in this study may in fact relate to mCRP induced effects rather than pCRP.

### Murine Fibrosarcoma, Melanoma, Sarcoma, and Colon Adenocarcinoma

Deodhar et al. (29) used the Fibrosarcoma T241 cell line to study the anti-metastatic activity of CRP in a murine xenograft model. Tumor was implanted on one hind foot and, after 17 days, the primary tumor was excised. CRP was prepared in liposomes and injected intravenously. CRP/liposome-treated animals had significantly fewer and smaller metastases than control animals. Furthermore, it was better to give CRP early during malignancy to prevent metastatic growth than to give CRP after the metastatic site formed. Thirty-eight percent of the CRP/liposome-treated animals were completely free of metastases compared to 0-2% of controls. The authors point out that CRP had to be encapsulated in liposomes since free, non-encapsulated CRP, given even at a 40-times higher dose, was ineffective.

Using monoclonal reagents (49, 50) and biophysical analyses to identify mCRP in pCRP samples, the procedures used by Deodar et al. (29) to prepare CRP in liposomes were used to quantify and differentiate the pCRP and mCRP proteins. Large multi-lamellar liposomal encapsulated CRP was prepared in a 1:1, M:M mixture of Phosphatidylcholine (PtC): Phosphatidylserine (PtS) which was dried onto a surface prior to adding buffered CRP in a nitrogen-filled tube and vortexing for 1 min. Of that CRP which incorporated into liposomes, 42% expressed mCRP antigenicity (45, 44). If a similar conversion between CRP and mCRP occurred when CRP-liposome reagents were prepared for the studies reported, the anti-metastatic effects attributed to CRP could, at least in part, have been due to mCRP. Additional independent analyses have now established that pCRP converts into mCRP when in proximity to apolar lipid membranous zones (especially when membrane lipids are activated into lyso-lipids [i.e., monoacylglycerophosphatidyl choline (MG-PtC)] which better allows pCRP to bind to its PC ligand (7, 21, 24, 48, 51).

When peritoneal exudate macrophages from mice were exposed to CRP/liposomes, tumoricidal activity was generated against syngeneic T241 fibrosarcoma and B-16 melanoma cells, and against allogeneic Sarcoma-1 cells (30). Peritoneal exudate macrophages collected from mice given CRP/liposomes *via* intraperitoneal injection, did demonstrate anti-tumor activity and enhanced oxidative metabolism. These studies suggested that CRP could be an effective immunomodulator in malignant disorders through its effects on macrophage function. In these studies, phagocytosis of IgG-coated liposomes partially inhibited phagocytosis of CRP/liposomes, supporting the concept that a CRP receptor is somehow associated with an Fc receptor on effector cells.

Thombre & Deodhar (31) extended these studies using a murine colon adenocarcinoma (MCA-38), which metastasizes to the liver. Mice receiving CRP/liposomes had significantly fewer and smaller liver metastases (25-28%) compared to animals in control groups (53-54%). As described above, liposomal entrapment of pCRP will cause pCRP to dissociate into the mCRP isoform (11).

Gautam et al. (32) and Gautam & Deodhar (33) reported that the tumoricidal effect against fibrosarcoma and colon carcinoma that CRP/liposomes had on peritoneal exudate cells was like the effect seen with muramyl-tripeptide (MTP)/liposomes except that the CRP/liposome effect required an intact complement system in experimental animals. Thus, the effectiveness of CRP in model systems of malignancies is like the effectiveness of CRP in infections in terms of complement requirements and involvement of mononuclear phagocytes.

### Murine Mastocytoma, Fibroblast Carcinoma, and Human CAK-1 Carcinoma

Zahedi & Mortensen (34) used CRP to activate mouse macrophages obtained from inflamed animals into a tumoricidal state. Exposing elicited macrophages to CRP for 30 min to 2 hours was sufficient to induce tumoricidal activity *in vitro* to the P815 mastocytoma cell line, the L-929 murine transformed fibroblast cell line, and the human CAK-1 carcinoma cell line. If CRP was removed from the culture medium using either anti-CRP reagents or affinity resins known to bind CRP, induction of tumoricidal activity was nullified. In these studies, CRP, but not CRP/liposomes, was used to stimulate inflammatory macrophages into a tumoricidal state. CRP-activated macrophages did not kill normal, explanted human fibroblasts. The effect of CRP did not appear to involve stimulation of T-lymphocytes to increase lymphokine production. Furthermore, CRP heat-aggregated at 85°C for 1 hour prior to measuring tumoricidal activity had significantly less killing activity than non-heat-aggregated CRP. It was reported that CRP bound to a subset of peroxidase-positive macrophages (30-35%) infiltrating a subcutaneous inflammatory site. Furthermore, binding apparently involved high affinity receptors, perhaps related to Fc receptors. It is unclear whether these results may have been influenced by mCRP that was present in the purified CRP reagent used for these experiments.

### Studies on the Mechanisms of CRP Tumoricidal Activity

Barna etal. (35) reported that CRP could activate human peripheral blood mononuclear cells to become tumoricidal *in vitro* against human astrocytoma (CCF-STTG1), renal carcinoma (CAKI-1), and melanoma (SK-MEL 28) cell lines. These studies used CRP that was not encapsulated in liposomes. Optimal tumoricidal activity required that CRP be incubated on adherent mononuclear cells for at least 20-44 hours (i.e., well beyond the 4-hour incubation period for conversion of pCRP to mCRP) and involved enhanced superoxide anion generation capacity. Significant tumoricidal activity was observed in 79% of the cell preparations taken from 24 normal individual donors. CRP did not affect normal, non-neoplastic human fibroblasts or glial cells. To see if at least part of the tumoricidal effect could be due to natural killer cell activity, adherent cells were treated with an antibody to natural killer cells and complement to effectively diminish the effect of these cells in the assay. No differences were noted suggesting the majority of the observed tumoricidal effects were mediated through macrophage activity. A later report, however, did state that anti-tumor activity elicited by CRP in liposomes could be abrogated by treating peritoneal exudate cells with anti-Thy 1.2 (reacting with a marker on T-lymphocytes) or anti-asialo Gm1 (reacting with a marker on NK cells) and complement (33). These later data suggest that further studies are needed to determine if the anti-tumor activities of CRP are mediated through effector cells other than macrophages.

The observed CRP effects described above were inhibited by preincubating CRP with PC ligand. Also, an unknown factor present in human serum, which was included as part of the cytotoxicity assay protocol, also inhibited the CRP-mediated tumoricidal effect. The relationship of this (these) factor(s) to CRP is somehow influenced by preincubating serum on microtiter plate-immobilized CRP and by the level of PC added to the test system. All these results are distinct from those observed when cytotoxicity was elicited with endotoxin thus suggesting the CRP effects noted both *in vitro* and *in vivo* are not due to contaminating levels of endotoxin in experimental preparations.

Barna et al. (36) extended these findings, showing that CRP could enhance tumoricidal activity of human alveolar macrophages. Interestingly, the CRP-stimulated cytotoxicity of macrophages collected from smoker volunteers was significantly depressed compared to non-smoker volunteers.

Overall, the tumoricidal activity of CRP in both *in vitro* and *in vivo* test systems suggest that “CRP” does have function as a biological response modifier in general reactions of macrophage function. In the systems investigated, CRP was found to be non-toxic to normal cells but to have vast potential as a non-specific agent against a variety of tumors, some of which are known to metastasize to various organs (52). These analyses need to be repeated using certified and distinctively separated pCRP and mCRP reagents so the true nature of “CRP” as a biological modifier in cancer can be advanced.

## Antitumor Effects of Peptides Derived From the Structure of CRP

Deodhar et al. (37) extended their studies of antitumor effects of CRP/liposomes to include novel peptides derived from the primary structure of CRP. At least one peptide (a dodecapeptide of residues 174-185 (IYLGGPFSPNVL) was found to demonstrate significant anti-tumor effects when administered to both metastatic and primary tumor murine model systems described in other studies. Peptide administered alone was found to be ineffective. In studying the mechanism by which CRP-peptide/liposome inhibits lung metastasis of murine fibrosarcoma T241, Barna et al. (53) demonstrated enhanced infiltration of MAC 1+ cells (now described as Complement-receptor 3 (CR3) positive cells) into affected lung. Peripheral blood MAC 1+ cells did not increase in number suggesting the increased number of immune cells at the tumor site was due to stimulation of directed migration at the site of disease.

Further efforts to identify the mechanism of CRP’s effects focused on how CRP influenced cytokine production. CRP was found to increase both interleukin-1 (IL-1) and tumor necrosis factor (TNF) secretion in both normal human monocytes and alveolar macrophages (40). CRP, or its active peptide-fragment, when encapsulated in liposomes, were maximally effective in protecting not only metastases in a murine fibrosarcoma model, but increased survival when liposome-encapsulated CRP (or its peptide) were injected in combination with interleukin-2 (IL-2) (39). Lung macrophages from CRP-peptide/liposome treated mice showed enhanced levels of TNF-α secretion with no apparent effect on interferon secretion (38). CRP also influenced IL-1β, IL-6 and TNF-α secretion of normal human monocytes grown in culture. In this latter study, the dose of CRP used was found to differentially influence the levels of cytokine production. Both IL-1β and IL-6 secretion increased with increasing levels of CRP while TNF-α levels peaked at a dose level of 50 ug/ml CRP and decreased when 125 µg/ml was used (42). Cells most influenced by CRP-peptide in tumoricidal responses implicated monocytes and not natural- or lymphokine-activated killer cells. As in *in vivo* experiments, monocyte responsiveness was best when CRP-peptide was used in combination with IL-2 (41). While CRP-peptide increased tumoricidal activity of both normal monocytes and alveolar macrophages, the mechanisms of enhancement were not necessarily identical since CRP-peptide only influenced TNF-α and IL-1β secretion from normal monocytes (43).

These data show that CRP effects in anti-tumor responses required prolonged incubation, the admixing of CRP with lipids, and/or the fragmentation of the intact CRP into reactive peptides; the likelihood is high that the mCRP isoform played at least some if not the predominant role in these reported anti-cancer activities. In this context, CRP, as a key acute phase reactant, can naturally influence the activity of the natural (non-specific) arm of the immune system, but that to elicit its powerful biological modifier response, requires a conformational change at localized tissue sites involved with disease.

## Early Studies Directly Comparing pCRP and mCRP in Animal Models of Cancer

*In vivo* experiments were performed to determine whether mCRP demonstrates anti-tumor activity in female BALB/c mice against a murine breast adenocarcinoma solid tumor (45). EMT6 breast adenocarcinoma cells were grown in culture and injected subcutaneously proximal to the calcaneus of the right hind limb of test animals. Once tumors were growing and palpable (5-to-7 days after implantation), approximately 100 µg mCRP (5 mg/kg) was injected intravenously through the tail vein every other day for at least 7 days. The activity of mCRP was evaluated by examining primary tumor growth and metastases, with observations made of tumor necrosis, appearance characteristics of each mouse, deaths, and, at necropsy, histology and immunohistochemistry of both tumor and non-tumor tissues.

To directly compare pCRP and mCRP effects, and to assess the effects of lipid vesicles on anti-tumor effects, Kresl et al. (45) prepared not only the soluble/self-aggregated form of mCRP, but mCRP and pCRP in specially prepared lipid vesicles called Large Unilamellar Vesicles or “LUVETs”. LUVETs are distinct from multilamellar vesicles in that 1.) they do not contain traces of organic solvents and detergents commonly found in liposome formation techniques, 2.) they are generated under physically mild conditions involving an extrusion procedure through a polycarbonate membrane under low pressure, 3.) they do not use vortexing to resuspend CRP into lipid vesicles, and 4.) they are defined by a single bilayer and contain a relatively large internal volume for greater encapsulation efficiency. Importantly, the procedures used to make LUVETs were chosen to limit the denaturation/dissociation of pCRP used in these studies.

Kresl et al. (45) demonstrated that while 42% of pCRP was converted to mCRP included in multilamellar vesicles, <1% of pCRP included in LUVETs converted to mCRP. Furthermore, when mCRP was included in LUVETs, no pCRP antigenicity was observed indicating that no renaturation to pCRP occurred in the lipid environment.

In the murine breast adenocarcinoma model, each of isolated and LUVET-encapsulated pCRP and mCRP were used to study effects on primary tumor cell growth and metastasis. Protein was administered by intravenous injection of 100-200 µl test agent (containing ~ 100 µg CRP protein) every second day for 14 days, beginning on the first day actual tumor could be palpated on the flank of the mouse (day 7). Seven days after tumor implantation, mean group tumor mass varied from 459 mm^3^ to 863 mm^3^. Tumors in mice receiving pCRP-LUVETs continued to grow at a rapid rate paralleling and not significantly different from that rate observed with mice in the no therapy control group. In contrast, tumor growth rate in mice receiving mCRP-LUVETs was minimal throughout this time-period and was significantly different from tumor growth in the no therapy control group.

Injecting mCRP without prior incorporation into LUVETs (i.e., mCRP-buffer therapy) was also effective in preventing growth of murine breast adenocarcinoma tumors. These data indicate that mCRP, and not pCRP, is biologically active *in vivo* as an anti-cancer agent. Visual examination of tumors on day 7 indicated the presence of a raised, subdermal solid tumor mass with well-defined borders having uniform color and texture over the entire surface of the tumor. After injecting mCRP, necrotic lesions (defined as a blackening of the skin surface with possible involution of tissue) was observed in at least 2/3^rd^ of treated mice while no mouse in any control group (n = 30 mice) showed signs of necrosis. Of note, one mouse (of 15) injected with pCRP-encapsulated LUVETs showed necrosis (suggesting the processes of preparing pCRP encapsulated vesicles could have produced some level of conversion of pCRP into mCRP). Necrotic lesions were soft and pliable to palpation and were well defined subdermal marks covering up to one-third of the entire tumor surface. Fine needle aspiration biopsy at one necrotic site verified dead tumor cells and a preponderance of polymorphonuclear leukocytes and macrophages at the site. Also, analysis of the biopsy indicated the observed necrosis was not the result of an infectious process.

At necropsy, discrete focal necrotic lesions were discovered within the tumor mass not visible by surface examination. No other organs or tissues were found to be abnormally affected by mCRP therapy indicating the anti-tumor mCRP effect is localized to the tissue-based pathology and does adversely affect other organs and tissues.

Deodhar et al. (29, 37) and Barna et al. (54) reported anti-cancer CRP reagents were effective at preventing metastases and death. The studies by Kresl et al. corroborate these findings but extend the understanding to emphasize that the conformation of CRP is a critical factor in eliciting the anti-metastatic effect. While 67% of control animals receiving no therapy or buffer controls showed signs of metastasis, only 40% of mice receiving mCRP-buffer therapy, and 6.25% of mice receiving mCRP-LUVET therapy demonstrated lung metastatic tumors. Together, these data indicate that “CRP” can have anti-cancer activity but that this biological activity is specific to the mCRP isoform.

## Discussion and Conclusion

The message shared in this overview is that the widely known innate immune system protein known as the prototypic acute phase reactant does have bioactivity that can enhance the natural host defenses against cancer. The *in vitro* and *in vivo* data generated and reported on in the literature is insightful, relevant, and contributes important insight into how CRP is and can be useful as both a natural, and a therapeutic agent in combating cellular overgrowth that occurs as part of cancer disease. The key new message related herein, however, is that that the interpretation of CRP’s effects must include awareness and control for the bioactivities of at least two conformational isoforms of CRP: 1.) the hepatically-produced serum soluble cyclic pentameric disk (i.e., pentameric or “pCRP”), and 2.) a non-proteolytically dissociated, conformationally modified, poorly soluble, lipid-inserting isoform with distinctive antigenicity (i.e., modified, monomeric CRP or “mCRP”.) While the experiments and results summarized in this report need to be repeated using clearly defined and certified CRP isoform reagents, based on the evolving understanding of the structures, bioactivities, biodistributions and antigenicity of CRP, a refined interpretation of published work does suggest that the mCRP isoform, rather than the pCRP isoform, contributes to anti-cancer immune and inflammatory responses. The effects appear to involve leukocytes, in particular monocytes/macrophages, and include stimulating enhanced oxidative metabolism and regulation of cytokine secretion (30, 55, 56). Specific peptides generated from the CRP subunit, identified as the cell binding peptide, could inhibit CRP’s effects on leukocytes (57). This peptide (residues 27-38 - TKPLKAFTVCLH) includes one of the cysteine residues involved in the sole intrachain disulfide bond of each CRP subunit, which is now known to overlap the cholesterol binding sequence reported for mCRP (48, 58). Of importance, full exposure of the cholesterol binding sequence to maximize mCRP insertion into cholesterol-rich lipid rafts requires reduction of this intrachain bond (59). Indeed, the role of disulfide bonds as allosteric modulators of protein function is an evolving concept of widespread biochemical relevance (60) Intramolecular stress contributed by covalently bonded disulfides, which introduce a potential energy of activation in a protein, would be released upon reduction, causing the protein to re-distribute tertiary and quaternary biochemical energies and provide kinetic energy needed to elicit a productive biological response. Protein conformational changes of homo-polymeric proteins (which describes pCRP) can occur when the polymeric protein dissociates, changes conformation, and reassociates into a different quaternary complex (described as “Morpheenins” or “Transformers” (61–64); these changes affect and regulate protein function.

The mCRP isoform spontaneously forms from the pCRP isoform when the non-covalently associated pentamer dissociates. The interface between subunits that hold the pentamer together are stabilized by both apolar and electrostatic forces, and the globular CRP subunits are stabilized by the two calcium ions know to bind each subunit. In addition to stabilizing tertiary and quaternary CRP structures, calcium ions are necessary for CRP binding to its primary ligand – PC (65, 66). Calcium binding controls CRP flexibility around the PC binding sites (67) and, when present at 1 mM and above, protects CRP from proteolysis (8, 50, 68). However, when exposed to strong apolar surfaces such as the interior of a membrane bilayer, a plastic ELISA-plate surface (e.g., polystyrene), or an air-water surface interface (as would be enhanced by vortexing procedures), the calcium stabilization effect of the CRP pentamer is lost (7, 8), resulting in structural changes that profoundly affect CRP solubility and epitope expression (19, 20, 49). While the aqueous solubility of modified CRP decreases, some protein reassembles into self-associated multimers (as has been described for Morpheenins/Transformers), which remains soluble or suspended. The level of protein remining in aqueous phase is highly dependent on the ionic strength, pH, and the presence of divalent cations in the solvent (20) (**Table 1**). Because the conformational change of pCRP into mCRP does not require proteolysis, and since some level of soluble/suspended mCRP remains in an aqueous phase, the presence of mCRP in a purified “CRP” test reagent would not be immediately apparent. Indeed, both pCRP and mCRP subunits would be indistinguishable using SDS-PAGE as a purification criterion. In assays preformed directly comparing the distinctive bioactivities of pCRP and mCRP, mCRP is found to be 5-10-fold more potent (5, 8, 21, 46, 69, 70). Concentrations of 1-10 µg/ml elicit strong effects in various *in vitro* assays. It is noted throughout the literature, it is often necessary to add 100-200 µg/ml of purified “CRP” reagent to an experimental system to see the CRP effect. Such levels are rationalized as representative of a robust CRP acute phase response. However, if the mCRP was present at 1-10% of the CRP used in such studies, the effect often attributed to the perceived pentameric protein might really be due to the 1-10 µg of the mCRP “hidden” in the reagent used.

Another important note related to the reliability of CRP reagents used in CRP experiments relates to the antigenic specificity of polyclonal anti-CRP reagents used to assess and certify antigenic identity of CRP. Most, if not all polyclonal antisera to “CRP” have been shown to have specificity to both antigens, quantified at up to 33% specificity to mCRP in analyzed “anti-CRP” reagents (8). By carefully reviewing published methods, one may postulate on the isoform specificity of CRP relevant to the results reported. If a low dilution of polyclonal anti-CRP was needed to generate reported results (e.g., 1/100), one can infer this reflects on a lesser specificity in the antiserum reagent (e.g., the mCRP isoform); if a high dilution was sufficient (e.g., 1/1000 or 1/10,000), one can infer the biospecificity being assessed is for the predominant isoform present (i.e., pCRP). As monoclonal antibodies have become available, there remains some confusion as to which CRP isoform is being evaluated. Monoclonal anti-CRP Clone 8 – used by many laboratories, is, in fact, specific for mCRP and not the pCRP isoform (71). Most interpretations of data involving use of Clone 8 do not discuss the presence or possible relevance of CRP isoforms in the interpretation of shared data.

The strongest reproducible data on CRP bioeffects in both *in vitro* and *in vivo* cancer models required incorporating the CRP reagent into lipid vesicles. Numerous studies emphasize how the conversion of pCRP to mCRP involves insertion into membrane lipids, especially in association with cholesterol, an interaction that is enhanced if the intra-subunit disulfide bond in the CRP subunit is reduced. When mCRP enters membranes, it triggers strong pro-inflammatory responses that, in consideration of observed anti-cancer effects described in various studies, suggest mCRP has favorable therapeutic activities (21, 24, 51). Lipid-associated mCRP particles (i.e., in micro vesicles) have been measured in patient serum after the onset of an inflammatory reaction associated with disease (25, 51, 72, 73).

The take-home message presented herein is that CRP does have anti-cancer bioactivities. While some laboratories trying to reproduce published studies may have concluded that CRP is not biologically relevant, considering an awareness of distinctive isoforms of CRP that are difficult to recognize in purified reagents and that have vastly different bioactivities, a consistent explanation for CRP’s role emerges. The mCRP isoform is now known as a strong pro-inflammatory signal. In a murine breast adenocarcinoma model, mCRP either in lipid vesicles or in buffer, significantly reduced tumor growth over the therapy period (45). Necrosis was noted at the tumor site in most treated mice, and fewer pulmonary metastatic lesions were seen in mCRP-treated animals. The role of CRP as an anti-metastatic agent is of intrigue.

In a murine fibrosarcoma pulmonary metastasis model (29), all untreated animals died within 40 days of tumor implantation with confluent pulmonary metastases. Twenty percent of animals receiving a CRP-multilamellar vesicle (MLV) mixture survived to at least day 90. Animals surviving to day 180, when examined at necropsy, were found to be free of recurrent or metastatic disease. This group extended these studies to co-administer Interleukin-2 with lipid-encapsulated CRP. All untreated animals died by day 40 with large numbers of pulmonary metastatic lesions. While 13-23% of IL-2 therapy-alone-treated animals survived to day 90, and twenty percent of CRP-MLV alone-treated animals survived the same 90-day period, the combination of IL-2 and CRP-MLV therapy resulted in greater than 50% of animals surviving this same 90-day experimental period. Surviving animals showed essentially no metastatic lesions at necropsy, and when lungs were examined immunobiologically, more Thy 1.2 cells were found suggesting the anti-metastatic effect observed in these animals may involve enhanced T- cell immunity.

In a murine colon adenocarcinoma liver metastasis model, 41% of control animals survived to day 180, while 77% CRP-MLV-treated animals survived over the same period. Liver metastases were found in all animals dying over the experimental period; long-term survivors were free of tumors (32).

A similar study using a murine renal carcinoma model, peritoneal cells collected from liposome encapsulated CRP slowed tumor cell growth in naïve mice (74). Macrophages were the predominant cell found in the peritoneal effusion. In subsequent experiments, cells collected from the lungs of CRP-MLV-treated mice (also predominantly macrophages), were found to inhibit the growth of tumor cells *in vitro*, suggesting that the anti-tumor effect of CRP observed in these experiments occurs predominantly by stimulating the macrophage cell population.

A primary component of the CRP effector response against tumors is the monocyte/macrophage (30, 34, 35, 38, 40, 41, 43). Active CRP reagents increased production of reactive oxygen and nitrogen species and increased secretion of cytokines such as IL-1β and IL-6 (40). As many of these studies involved incubating CRP in tissue culture for 24-72 hrs., a condition now known to promote the dissociation of pCRP into its mCRP conformer, it is reasonable to question whether results generated may have been propagated by the mCRP rather than the assumed pCRP conformer. When mCRP is produced *ex vivo* and added directly to tissue culture systems, it is shown to be active in the first hours after addition and to elicit responses at much lower concentrations as were needed when pCRP reagents were used in reported studies. mCRP has been shown to activate endothelial cells, to promote neutrophil adhesion to endothelial cells, to activate neutrophils and delay their apoptosis, to increase neutrophil synthesis and secretion of IL-8 and to activate platelets (7, 46, 47, 59, 69, 70, 75). *In vivo* murine models, CRP, both alone and in combination with lipid vesicles, is shown to inhibit tumor growth and metastasis (45).

CRP was first identified and named by Abernethy & Avery (76) as a protein that appeared in blood during the acute phase of pneumococcal infection. Its presence, and dynamic quantitative levels as a protein associated with any disease or condition involving tissue damage and the associated inflammatory response, has been recognized and studied for decades. While its clear quantitative association with inflammation has enabled it to be a useful diagnostic marker, its biological, mechanistic role in activating and regulating host defense reactions has eluded definition for over a half-century. By introducing CRP as a protein that can and does undergo a pronounced conformational transformation, its biological role as an activator and amplifier of inflammation can be described in responses relevant to the natural host defense against cancerous growth. Through recognizing the relationship and differences between its pentameric, serum soluble conformer (i.e., pCRP) and its modified, monomeric, poorly soluble, lipid-binding conformer (i.e., mCRP), a consistent explanation of CRP’s bioactivity is advanced.

## Author Contributions

LP – summarized the literature with special insights on different CRP isoform structures and functions, and wrote the manuscript. PH – edited the manuscript and contributed to the use and utility of cancer models used. IR – edited the manuscript and contributed details related to the structure and function of CRP isoforms. MO – edited the manuscript for contact and clarity and provides insights into peptide reagents and model systems used. All authors contributed to the article and approved the submitted version.

## Conflict of Interest

The authors declare that the research was conducted in the absence of any commercial or financial relationships that could be construed as a potential conflict of interest.

## Publisher’s Note

All claims expressed in this article are solely those of the authors and do not necessarily represent those of their affiliated organizations, or those of the publisher, the editors and the reviewers. Any product that may be evaluated in this article, or claim that may be made by its manufacturer, is not guaranteed or endorsed by the publisher.

## Abbreviations

pCRP, pentameric discoid CRP; mCRP, monomeric, modified CRP (the conformationally distinctive CRP isoform expressed when pCRP subunits dissociate); PtC, Phosphatidyl Choline (the lipid); Lyso-PtC, monoacyl phosphatidyl choline; PC, Phosphocholine (the hapten); PtS, Phosphatidylserine; APR, Acute Phase Response; LUVETS, Large Unilamellar Vesicles made by an Extrusion procedure.
