# Supplementary material for: C-Reactive Protein and Cancer: Interpreting the Differential Bioactivities of Its Pentameric and Monomeric, Modified Isoforms
Source: Front Immunol. 2021 Sep 6;12:744129. doi: 10.3389/fimmu.2021.744129 (PMC8450391; doi:10.3389/fimmu.2021.744129)
Supplement: Supplementary file 1 [file DataSheet_1.docx]

**Supplementary Figure 1:**

**Schematic depiction of the biomechanistic interrelationship of pCRP and mCRP isoforms**


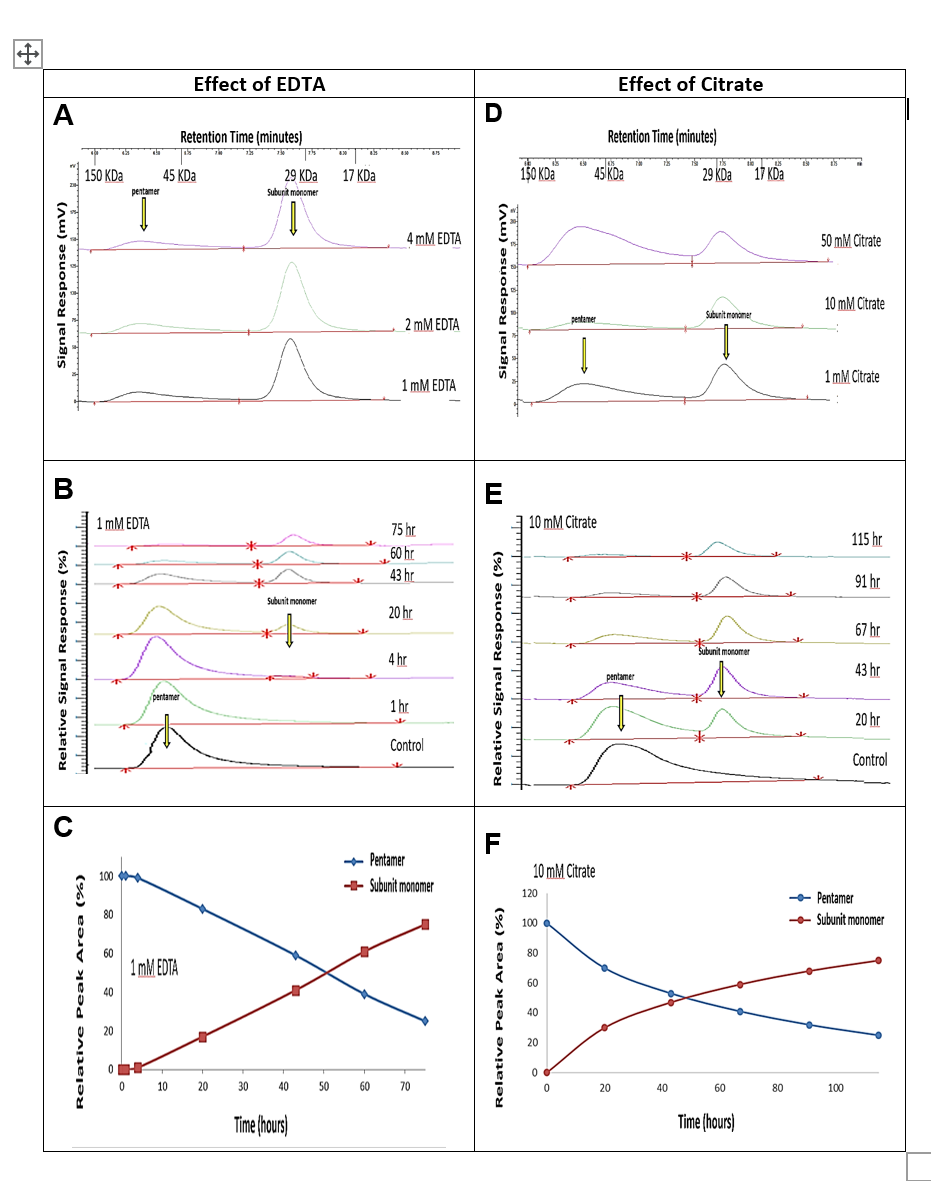


Panel A, B and C: pCRP (final concentration 20 μg/ml) was diluted in 75 mM Tris-HCl, 0.15 M NaCl (Tris-saline) pH 7.4 and incubated at 37 ⁰C with indicated EDTA concentrations and time intervals. 1μl of each sample was injected and analyzed on a Shimadzu Prominence HPLC system by size-exclusion HPLC analysis using an Agilent AdvanceBio 300 x 7.8mm column at 27 ⁰C. Protein was detected using intrinsic fluorescence at Ex 292nm and Em 355nm.

Panel A compares the effect of different EDTA concentrations. Panel B compares the time course of incubation using 1 mM EDTA; Panel C compared the relative peak area of the curves generated for pCRP (eluting with an apparent Mw of 115,000) and mCRP (eluting with an apparent Mw of 23,000). The molecular weight calibration curve was established using IgG 150 kDa; Chicken Albumin 45 kDa; Carbonic Anhydrase 29 kDa; and Myoglobin; 17 kDa.

Panels D, E and F: pCRP was similarly prepared and analyzed as above except calcium chelation was achieved using 75 mM Tris HCl, 0.15M NaCl (pH 6.0) with indicated citrate concentrations. Results show dissociation of the pentamer into a monomer occurs within hours when calcium remove by either chelating agent. Chelation with BAPTA and Chelex 100 produced similar results as shown.

Panel D compares the effect of different citrate concentrations. Panel E compares the time course of incubation using 10 mM Citrate; Panel C compared the relative peak area of the curves generated for pCRP and mCRP.

Conditions used were adapted from experiments described in Potempa et al. 1987.

**Supplementary Figure 2:**

**Preliminary data showing the effect of intravenously injecting recombinant modified CRP into A.) a murine model of Lewis Lung Carcinoma and B.) a murine model of human pancreatic CAPAN-2 tumors**

In *in vivo* experiments similar to those described in Kresl et al. (1999), which used a biologically isolated and produced form of mCRP, a recombinant form of mCRP (r_m_CRP) (Potempa et al. 2015) was analyzed for anticancer activity in mouse models of disease. After tumors were implanted and palpable, r_m_CRP was injected intravenously at 2.5 mg/kg every other day for seven days. Tumor mass and necrosis was monitored in preliminary studies. Panels A show the effect of r_m_CRP injections in a Lewis Lung tumor model in Balb/c mice. Panels B show the effect of r_m_CRP injections in a human pancreatic CAPAN-2 tumor model in nude mice. As in the data described by Kresl, using the EMT6 murine breast adenocarcinoma model, the recombinant analog of human mCRP was effective at slowing tumor growth and causing necrosis at the site of tumor implantation in both a murine lung cancer model and in a human pancreatic cell model in nude mice. Modified CRP bioactivity in mice lacking an effective T-lymphocyte immune response is consistent with its reported stimulatory effect on leukocyte responses. Mouse experiments were performed by Battelle Laboratories (Columbus, Ohio; study Number N003959A) and conformed to AAALAC standards, NIH Guidelines for the Care and Use of Laboratory Animals, and the US Department of Agriculture Animal Welfare Act.
